# Motor vehicle collisions caused by the ‘super-strength’ synthetic cannabinoids, MAM-2201, 5F-PB-22, 5F-AB-PINACA, 5F-AMB and 5F-ADB in Japan experienced from 2012 to 2014

**DOI:** 10.1007/s11419-017-0369-6

**Published:** 2017-05-29

**Authors:** Shuji Kaneko

**Affiliations:** Department of Molecular Pharmacology, Graduate School of Pharmaceutical Sciences, Kyoto University, Yoshida-Shimoadachi-cho 46-29, Sakyo-ku, Kyoto, 606-8501 Japan

**Keywords:** Synthetic cannabinoid, Motor vehicle collision, Catalepsy, Anterograde amnesia, Intoxication symptoms

## Abstract

From 2012 to 2014 in Japan, 214 cases of motor vehicle collisions were attributed to the use of illegal drugs. In 93 out of 96 investigated cases, the causative agents were a variety of synthetic cannabinoids (SCs). These SCs can be classified into three groups according to the lineage of the chemical structures: (1) naphthoyl indoles, such as MAM-2201, (2) quinolinyl ester indoles, such as 5F-PB-22, and (3) indazole carboxamides, such as 5F-AB-PINACA, 5F-AMB, and 5F-ADB. These SCs became available sequentially with increasing cannabinoid CB_1_ agonist potencies and reached a nationwide outbreak in the summer of 2014. They caused acute intoxication with impaired consciousness, anterograde amnesia (impaired memory), catalepsy with muscle rigidity, tachycardia, and vomiting or drooling soon after smoking. Drivers who had abused one of these SCs might unexpectedly experience the acute intoxication that caused uncontrolled driving. These SCs were generally difficult to detect from body fluid samples. It is thought that the highly lipophilic SCs disappear from the blood via rapid degradation by liver enzymes and selective accumulation into adipose tissues. Thus, much effort should be directed to the development of fast and sensitive chemical detection of the drug usage.

## Introduction

Synthetic cannabinoids (SCs) are a diverse class of compounds that are designed to elicit selective agonist activities on cannabinoid CB_1_ and/or CB_2_ receptors. Originally, SCs were developed in an attempt to find potentially safer therapeutic agents. However, many SCs have recently emerged that possess new chemical structures, and are widely abused as recreational cannabis substitutes [1]. The outbreak of SCs has social ramifications, particularly relating to the increased number of motor vehicle accidents, which occur easily since SCs are inadvertently and handily used by drivers when they smoke herbal mixtures containing SCs. The hazardous effects on driving are evident from the inhibitory actions of SCs on the central nervous system, resulting in somnolence and retarded movements. In addition, other psychotropic and physical adverse effects of SCs [1, 2] may be involved in causing motor vehicle accidents.

In Japan, 214 motor vehicle collisions were attributed to the use of illegal drugs from 2012 to 2014 (as announced in an official Japanese document from the National Police Agency). The number of suspected cases was 19 in 2012, 38 in 2013, and explosively increased to 157 in 2014. Accordingly, the Japanese Government has tightened the regulations on illegal drugs, and the outbreak has apparently been suppressed from 2015 to the present. The investigative organizations have provided the author with evidence on 96 serious cases that took place from 2012 to 2014. Most of the suspected compounds were SCs, although it was difficult to establish a causal relationship between drug use and an accident because of the lack of human toxicological or pharmacokinetic data on newly identified compounds. Moreover, only limited data were available for laboratory tests in these cases, because blood testing requires a court-issued warrant in Japan, which is difficult and time-consuming to obtain. However, the timeline of detected substances, drivers’ statements, and observed symptoms were useful for a better understanding of the detrimental effects of SCs on driving. In this review, the author summarizes the toxicological features of recent ‘super-strength’ SCs by grouping compounds found in the 96 cases and highlighting typical accidents.

## Overview

Table 1 summarizes the observational data on the 96 investigated cases. All accidents were caused by male drivers with an average age of 30.8 years, reflecting the distribution of illegal drug abusers in Japan. The accidents occurred mainly in metropolitan areas in 2012, but afterward spread to rural areas where automobiles are required for daily living. Four persons were killed in four tragic cases, and more than 110 persons were injured in 51 cases. The objective appearances of the drivers after the collision were collectively described as ‘impaired consciousness’ in 73 out of 96 cases (76%), but excited states such as agitation, shouting, confusion, and continuous stereotyped behaviors were reported in 16 cases (17%). The causative agents were mainly hypothesized to be chemical compounds detected in the drivers’ belongings, such as pipes and herbal pieces. In 93 out of 96 cases, one or more SCs were found, sometimes in combination with cathinones or diphenidines. Only diphenidine or its derivative was detected in the remaining three cases. There was no case in which only cathinone derivatives were used. These results suggest that SCs were the primary cause of motor vehicle collisions in most of these cases.

**Table 1:** Overall characteristics of drug-induced motor vehicle collisions in the 96 investigated cases that occurred during 2012–2014 in Japan

Properties	*n*
Driver’s gender	
Male	96
Female	0
Driver’s age - Range 19–54	
Mean	30.8
Median	30
Accident classification	
Fatal	4
Injury	51
Property damage	41
Appearance just after the collision	
Impaired consciousness	73
Excited or confused	16
Unknown	7
Used (or possessed) substances	
One SC	43
Multiple SCs	30
SC + cathinones	14
SC + diphenidines	6
Diphenidines only	3
Cathinones only	0

*SC* synthetic cannabinoid

However, only limited data were available about blood and urine concentrations of SCs in these cases (Tables 2 and 3), indicating that direct evidence of illegal SC use was obtained in only one third of all cases. Of the 20 cases in which SCs were used in combination with other psychostimulants, cathinones or diphenidines were detected in 12 cases, while SCs were identified in only five cases. These facts suggest the difficulty of detecting and profiling SCs in biological samples, because of the relatively small amount of intake, unknown metabolism, and possible instability in the human body.

**Table 2:** SCs identified in blood samples

Name	*n*	Concentration
AM-2232	1	N.Q.
5F-PB-22	6	Range 0.274–0.93 ng/mL (*n* = 5), median 0.35 ng/mL
FUB-PB-22	4	Range 0.79–1.74 ng/mL (*n* = 2)
NM-2201	1	N.Q.
AB-PINACA	1	0.04 ng/mL
NNE-1	1	2.3 ng/mL
5F-AB-PINACA	1	0.16 ng/mL
AB-FUBINACA	1	N.Q.
5F-AMB	4	0.07 ng/mL (*n* = 1)
AB-CHMINACA	4	1.3–31 ng/mL (*n* = 3), median 2.7 ng/mL

*N.Q.* not quantified

**Table 3:** SCs identified in urine samples

Name	*n*
FUB-PB-22	1
NNE-1	1
5F-AB-PINACA	3
5F-AMB	7
AB-CHMINACA	3
5F-ADB	2

## Lineage and timeline of SC

**Fig. 1:**
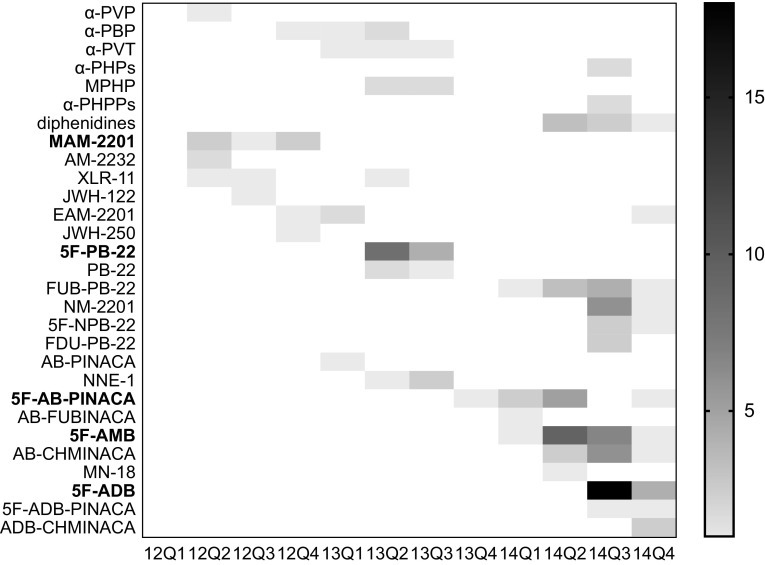
Heat map of illegal drugs found in the investigated cases of motor vehicle collisions during 2012–2014 in Japan. α-PHPs includes α-PHP and its 4-fluoro derivative. α-PHPPs includes α-PHPP, its 4-fluoro, and 4-methoxy derivatives. Diphenidines includes diphenidine and its 2-methoxy derivative. For the* horizontal axis*, the time span is indicated; for example, “12Q1” means the first quarter of 2012. The *right vertical bar* with *different densities* shows the number of cases

Figure 1 shows the quarterly timeline of illegal compounds detected over 3 years. In total, ten cathinones, two diphenidines, and 22 SCs were identified. Assuming that SCs are the principal cause of impaired driving, the timeline can be divided into three sections according to the common structure of the prevalent SC:

1. Until 2013 spring when naphthoyl indoles, such as MAM-2201, were dominant.
2. Spring to winter 2013 when quinolinyl ester indoles, such as 5F-PB-22, were dominant.
3. 2014 when indazole carboxamides, such as 5F-AB-PINACA, became dominant.

The following chapters deal with the transition of SCs with reference to the 12 typical cases of accidents, in which observational consequences are well-documented and clearer than other cases (Table 4).

**Table 4:** Typical cases of SC-induced motor vehicle collisions

Case	yymm	Age	Case history	Symptoms	Laboratory results
1	1205	25	A driver smoked ‘Super Zeus’ twice 1.8 km before the collision. The car made a jump-start reaching 114 km/h at a crossing on a red-light. Two cars were damaged, and 5 persons were injured	The driver had blurred vision and a dull headache 0.9 km before the accident. After the crash, he looked excited with abnormal postures. Driver had no memory of the crash	MAM-2201 and AM-2232 were detected in the herbal mixture. AM-2232 was detected from the blood sample
2	1210	31	A high school girl was hit by a car with her bicycle on a crosswalk and died. There was no sign of braking. The road was simply straight	The driver confessed to the use of a herbal mixture while driving. He had a reddened face, looked agitated, and then ran away from inspection	MAM-2201 was detected in the herbal mixture
3	1304	41	While driving a sports car, the driver smoked a pinch of herbal blend.Then 5–10 min later, the driver was alerted by a horn. After 5 min, the car crashed into the narrow space between the leading vehicles. Five cars were damaged and six persons were injured	The driver looked emotionless. He kept flooring the accelerator pedal even after the crash, and the tires were melted down. Driver had no memory of the crash	5F-PB-22 and PB-22 were detected in the herbal material. Blood and urine samples were not taken
4	1306	24	Driver was a long-term abuser. On a one-lane roadway, his car suddenly went straight into the opposite lane. Two cars suffered frontal collisions, and two persons were injured	Driver lost consciousness. His eyes rolled up, and he made groaning sounds. Driver recovered after 3.5 h, but had no memory of the crash	5F-PB-22 (0.274 ng/mL) was detected in the blood sample taken 45 min after the car crash
5	1309	38	A driver had been using herbal drugs for two years. His car ran onto the sidewalk at a speed of 20 km/h and hit 4 persons without braking	Even after the crash, the driver did not step off the accelerator pedal, but kept on moving the steering wheel and gear lever in a stereotyped manner. Driver had no memory of abnormal driving	5F-PB-22 and NNE-1 were detected in the herbal material. Blood was sampled 17 h after the crash, and 0.39 ng/mL 5F-PB-22 was detected
6	1401	29	A driver received a parcel containing a herbal drug at a post office and immediately smoked in his car. Six minutes after smoking, his car went straight on a curve road at 36 km/h and drove into the opposite lane. A child walking on the opposite sidewalk after the school was hit and the child died	The driver kept grasping the steering wheel and did not respond to bystanders’ shouts. Driver had no interest in the car crash. Emergency crew identified traces of vomiting. After 25 min, the driver recovered and could walk by himself. He had no memory of the crash	5F-AB-PINACA was detected in the herbal mixture, but not in the blood sample taken after 49 h after the crash
7	1402	37	Two persons smoked herbal drugs while driving a sports utility vehicle. When a police patrol car followed their car at a red crossing signal, the car suddenly ran away into the narrow space between the leading vehicles. Ten cars were damaged and 12 persons were injured	The driver exhibited impaired consciousness with slow movements. His reaction turned to ‘crazy’ excitation and agitation during inspection by police. Driver had no memory of the crash but could recall the place of smoking	FUB-PB-22 (1.74 ng/mL) was detected from a blood sample taken 75 min after the crash. In addition, 5F-AMB, THJ-2201, and AMB were detected in the herbal mixture
8	1404	28	A driver was a long-term abuser. He ignored a red light at 80 km/h and caused successive vehicle collisions. Eight persons were injured	The driver was awake but motionless, and he did not respond for 10 min. He started moving 15 min after the crash and responded by talking thereafter. Driver had no memory of the crash	5F-AMB was detected in the herbal mixture and in the blood sample taken 14 h after the crash (but was not quantified)
9	1405	19	Two persons took turns smoking herbal blends while driving. At a crossing, the car suddenly jump-started into the opposite lane, reaching 100 km/h. The car drove more than 100 m and caused several head-on crashes one after another. One young man was killed and two persons were injured	Driver exhibited impaired consciousness with drooling and a heart rate of 123 beats/min. Driver had no memory of the jump-start but he remembered smoking. The passenger ran away	5F-AMB was detected in urine samples of the driver and passenger
10	1406	37	On a busy downtown street, a car drove on a wide sidewalk for 30 m and hit many walkers one after another until it was stopped by collision with a telephone booth. One young lady was killed and seven persons were injured	Driver lost consciousness and was drooling. He had no memory of what occurred after smoking	5F-AMB and AB-CHMINACA were detected in the herbal mixture. In addition, 5F-AB-PINACA was detected in the urine sample
11	1407	26	An unlicensed driver smoked while driving. Five minutes later, his car began weaving and was accelerated to 80 km/h. The car successively hit a motorbike and several cars, and it was stopped by collision with a lamppost on the sidewalk. Three persons were injured. The entire proceeding was recorded in a dashboard camera	Driver was found wandering around the car with impaired consciousness. He had no memory of driving from 2 min before the car started weaving	5F-AMB (0.07 ng/mL) and FUB-PB-22 (0.79 ng/mL) were detected in the blood sample taken 10 h after the crash. In the urine, 5F-AMB and THC were detected
12	1409	30	The driver was a long-term abuser for more than 3 years and never experienced memory loss. However, after a brief smoking of ‘Heart Shot Red’, he lost memory. Within 5 min, his car crashed into a parked car	Driver lost consciousness, but recovered within an hour. He had a heart rate of 106 beats/min with tachypnea 13 min after the crash	5F-ADB was detected in the urine, saliva and hair. 4F-α-PHPP was detected in the blood

*yymm* first two figures show the year and last two the month

### MAM-2201 and naphthoyl indoles

**Fig. 2:**
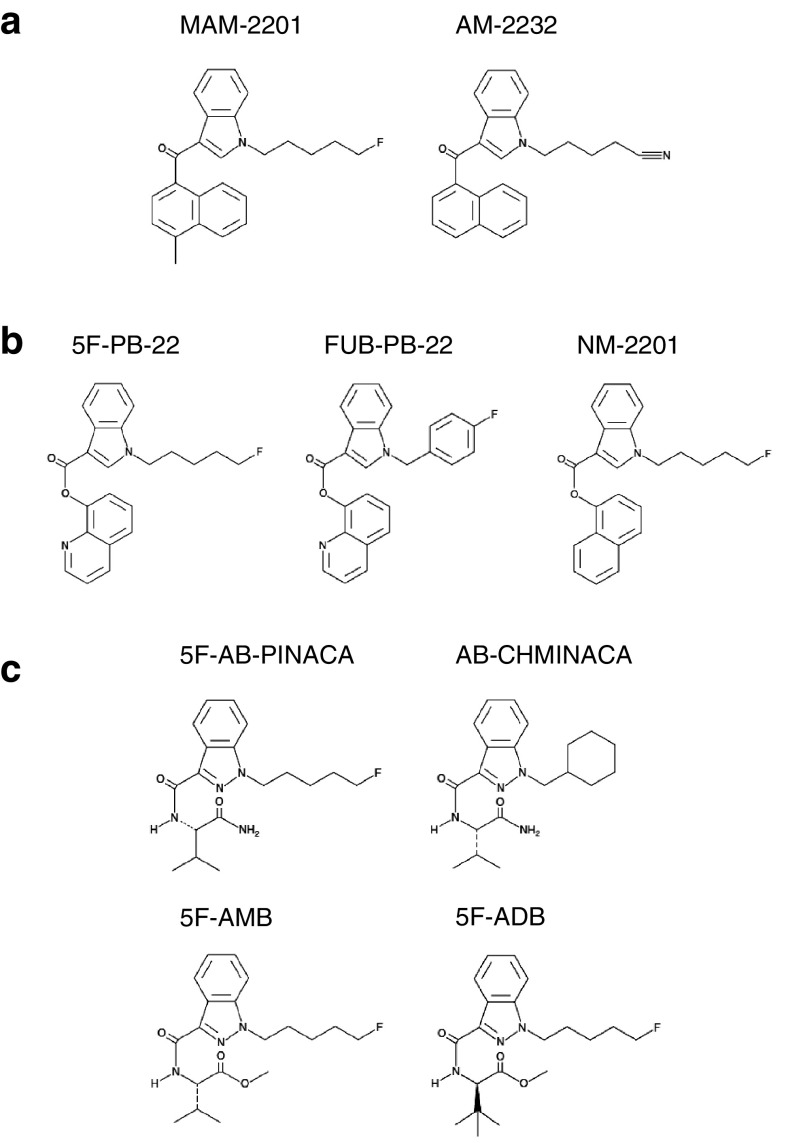
Frequently-used synthetic cannabinoids with **a** naphthoyl indole, **b** quinolinyl ester indole, and **c** indazole carboxamide backbones

The first-generation SCs were naphthoyl indole derivatives, such as JWH-018, which are known as the main component of ‘Spice’ and ‘K2’ drugs [2, 3]. Some of these SCs have more potent affinity and efficacy than the natural cannabis ingredient Δ^9^-tetrahydrocannabinol (THC), enabling stronger in vivo pharmacodynamic effects at lower doses [1]. The classical naphthoyl indole SCs were banned one-by-one as ‘designated substances’ by the Pharmaceutical Affairs Act in Japan. Accordingly, more diverse derivatives, such as MAM-2201 and AM-2232 (Fig. 2a) had emerged by 2012 [4], thereby evading the regulations.

These new naphthoyl indole SCs were involved in ten cases of motor vehicle collisions, often in combination with cathinone stimulants. However, as seen in the typical cases 1 and 2 (Table 4), naphthoyl indole SCs elicited excitation behaviors by itself, as reported in an intoxicated patient with MAM-2201 [5].

The naphthoyl indole SCs were barely determined in blood samples and never detected in urine samples. This may be due to the following reasons: (1) rapid action and rapid disappearance from blood, as reported in a controlled administration study of JWH-018 [6]; (2) high lipophilicity, as shown by a fatal case in which MAM-2201 was concentrated in the adipose tissue [7]; (3) low dose, according to their strong potency. As for the CB_1_ agonistic potency of MAM-2201, the IC_50_ value in inhibiting excitatory synaptic transmission in mouse cerebellum was 2.5- and 3-times lower than WIN-55,212 and JWH-018, respectively [8], demonstrating that MAM-2201 is stronger than the classical naphthoyl indoles. In addition, the mean concentration of MAM-2201 was reported to be 1.04 ng/g whole blood in Swedish recreational uses [9], indicating that only 2.8 nM blood concentration is sufficient to elicit cannabis-mimetic actions.

These naphthoyl indole SCs were banned by the structure-based, inclusive regulation of 2013 spring, and they disappeared from the Japanese illegal market.

### 5F-PB-22 and its analogs

Before the disappearance of naphthoyl indoles, a new type of SCs with quinolinyl ester indole and indazole carboxamide backbone structures emerged [10]. Among them, 5F-PB-22 (Fig. 2b) was involved in all 15 cases during the half-year from April to September 2013 until it was banned as a designated substance.

5F-PB-22 was 100 and 36 times more potent than WIN-55,212 and JWH-018, respectively, in a functional agonist assay using recombinant human CB_1_ receptor [11]. A long-term abuser who had been accustomed to naphthoyl indole SCs might become easily intoxicated after routine smoking of herbal blends containing the new ingredient that was stronger than before, as seen in the cases 3, 4, and 5 (Table 4). Catalepsy with muscle rigidity was the characteristic acute symptom in these cases, which may be the direct cause of accidents.

5F-PB-22 was detected from blood samples in six cases, and could be determined in five cases with a range of 0.274–0.93 ng/mL (Table 2). The average concentration of 0.45 ng/mL is equivalent to 1.2 nM, suggesting its strong toxicity. In four postmortem cases, the observed concentration range of 5F-PB-22 was 1.1–1.5 ng/mL [12], indicating that the toxic dose for driving is close to the lethal dose. Because 5F-PB-22 is highly lipophilic, and the predominant metabolic pathway of 5F-PB-22 in human hepatocytes is ester hydrolysis yielding a wide variety of metabolites [13], it is difficult to identify its unchanged form or metabolites in urine. Indeed, 5F-PB-22 was never detected in urine samples.

After 5F-PB-22 was banned, several derivatives of 5F-PB-22, such as FUB-PB-22 and NM-2201 (Fig. 2b) appeared and were used in 2014. Although these SCs were involved in a number of cases (Fig. 1), including cases 7 and 11 (Table 4), their pharmacodynamic and pharmacokinetic data are currently unavailable.

### 5F-AB-PINACA, 5F-AMB and their analogs

From the end of 2013, SCs with indazole carboxamide backbones have become predominant (Fig. 2c). This type of SCs was the strongest CB_1_ agonist and seemed to be designed and modified according to a patent applied by Pfizer [14].

5F-AB-PINACA was involved in 11 cases from 2013 December to 2014 April until it was banned. 5F-AB-PINACA is 38 and 360 times more potent than JWH-018 and THC, respectively, in a functional agonist assay using recombinant human CB_1_ receptor [15]. Accordingly, as seen in case 6 (Table 4), use of 5F-AB-PINACA while driving caused abrupt intoxication accompanied by catalepsy, vomiting, and loss of consciousness. Later, AB-CHMINACA, a similar compound with a cyclohexyl ring, appeared and was involved in 11 cases.

A nationwide outbreak of SC use occurred in the summer of 2014 with 5F-AMB and 5F-ADB featuring l-valinate and l-*tert*-leucinate, respectively, in the pendant group (Fig. 2c). 5F-AMB and 5F-ADB are 90 and 300 times, respectively, more potent than THC as full agonists for the human CB_1_ receptor [16].

5F-AMB was involved in 21 cases, including several tragic collisions such as cases 8–11 (Table 4). In serious cases, catalepsy was accompanied by muscle rigidity in the extremities, which provoked abrupt acceleration of the car without braking or controlling the steering wheel. Catalepsy is characterized in rodents as prolonged motionless at unnatural posturing and known as one of the tetrad of cannabinoid actions together with lowered body temperature, antinociception, and decreased spontaneous locomotion. These symptoms were also observed when mice were injected with AB-CHMINACA or AB-PINACA [17]. The rodent catalepsy recovers by external stimulus such as air-puff or sound, and they were reportedly mediated by decreased 5-HT transmission in the basal ganglia [18]. In contrast, in humans, strong SCs causes more severe, irreversible catalepsy with muscle rigidity, high body temperature, tachycardia, and vomiting. As these symptoms resemble the serotonin syndrome, the author and colleagues investigated the effect of 5F-ADB on midbrain serotonergic neurons. However, 5F-ADB did not affect serotonergic activity; rather, dopaminergic firings were readily increased [19]. Therefore, the neuronal mechanism underlying catalepsy seen in SC abuser is unclear.

The ‘final boss’ 5F-ADB was detected in the most frequent 24 cases, but fortunately, without a serious case. This may be because too much 5F-ADB was added to the herbal products, and drivers could not drive very far after smoking before losing consciousness, as seen in case 12 (Table 4). Accordingly, vomiting or a trace of vomiting was frequently observed in impaired drivers in 14 out of 23 cases involving 5F-ADB. It is also known that many people died in 2014 by smoking herbal products containing excess amounts of 5F-ADB (unpublished observation). Many news reports showing the dangerous effects of illegal drugs also helped to suppress the recreational uses. All these SCs were banned by the end of 2014.

An apparent difference in the action of this class of SCs was the time required for recovery from impaired consciousness. Users of naphthoyl indoles and quinolinyl ester indoles required more than an hour to communicate normally with other persons. However, drivers taking indazole carboxamide SCs frequently showed rapid recovery within an hour. It has been reported that 5F-AMB is quickly hydrolyzed by hepatic esterase [20], while 5F-AB-PINACA is more slowly metabolized to various products [21]. The rapid recovery seen in this class of SC remains mysterious.

Another characteristic of indazole carboxamide SCs is the detection from urine samples (Table 3). Increased polarity of the chemical structures may have contributed to the solubility of the compounds in aqueous solution. However, detection from blood samples was rare in this class of SC because the low dose and fast metabolism made the concentration in blood undetectable.

In a series of studies analyzing indazole carboxamide SCs in remaining human cadavers [22–24], 5F-AMB was mostly concentrated in the adipose tissue, while 5F-ADB and AB-CHMINACA were more widely distributed in other organs, such as brain and heart muscles. These SCs were never detected in the blood and urine, whereas MAB-CHMINACA (also called ADB-CHMINACA) was present in the blood as much as in other organs, although not in the urine. The mechanisms underlying distribution, metabolism, and excretion of SCs may be diverse and need to be investigated individually in detail.

## Concluding remarks

The most striking and almost common statements of SC-abused drivers are that they did not remember the collision scene, including before and after, although there may be some doubt on the drivers’ statements in themselves. The driver could drive properly (to some extent) with his eyes open while he could not remember what he was doing prior to the collision, suggesting that use of SCs caused anterograde amnesia that disabled new learning.

Most drivers seemed to be accustomed to using illegal drugs on some level and routinely used SC products while driving. However, the quality control of illegal products is so inaccurate and inconsistent that some packages might contain a huge amount of SC, as reported in the case of 5F-ADB [23], which might cause unexpected intoxication in a driver, instantly leading to uncontrolled driving.

Because the clinical signs observed in the abused drivers are transient in conjunction with the disappearance of active compounds from the body fluid, prosecution cannot be solely based on the material evidence. Much effort should be directed to the development of fast and sensitive chemical detection of drug usage.

The outbreak of SC is just like cyber-terrorism, wherein a wise scientist turns into a smart evil one. Given that the science extends its capability infinitely, we must keep in mind that there may be another outbreak of different kinds of illegal drugs.
